# Exercise preconditioning diminishes skeletal muscle atrophy after hindlimb suspension in mice

**DOI:** 10.1152/japplphysiol.00137.2018

**Published:** 2018-07-05

**Authors:** Nicholas T. Theilen, Nevena Jeremic, Gregory J. Weber, Suresh C. Tyagi

**Affiliations:** Department of Physiology, University of Louisville, Louisville, Kentucky

**Keywords:** exercise, mitochondria PGC-1α, skeletal muscle atrophy, TFAM

## Abstract

The aim of the present study was to investigate whether short-term, concurrent exercise training before hindlimb suspension (HLS) prevents or diminishes both soleus and gastrocnemius atrophy and to analyze whether changes in mitochondrial molecular markers were associated. Male C57BL/6 mice were assigned to control at 13 ± 1 wk of age, 7-day HLS at 12 ± 1 wk of age (HLS), 2 wk of exercise training before 7-day HLS at 10 ± 1 wk of age (Ex+HLS), and 2 wk of exercise training at 11 ± 1 wk of age (Ex) groups. HLS resulted in a 27.1% and 21.5% decrease in soleus and gastrocnemius muscle weight-to-body weight ratio, respectively. Exercise training before HLS resulted in a 5.6% and 8.1% decrease in soleus and gastrocnemius weight-to-body weight ratio, respectively. Exercise increased mitochondrial biogenesis- and function-associated markers and slow myosin heavy chain (SMHC) expression, and reduced fiber-type transitioning marker myosin heavy chain 4 (Myh4). Ex+HLS revealed decreased reactive oxygen species (ROS) and oxidative stress compared with HLS. Our data indicated the time before an atrophic setting, particularly caused by muscle unloading, may be a useful period to intervene short-term, progressive exercise training to prevent skeletal muscle atrophy and is associated with mitochondrial biogenesis, function, and redox balance.

**NEW & NOTEWORTHY** Mitochondrial dysfunction is associated with disuse-induced skeletal muscle atrophy, whereas exercise is known to increase mitochondrial biogenesis and function. Here we provide evidence of short-term concurrent exercise training before an atrophic event protecting skeletal muscle from atrophy in two separate muscles with different, dominant fiber-types, and we reveal an association with the adaptive changes of mitochondrial molecular markers to exercise.

## INTRODUCTION

Skeletal muscle atrophy results in the reduction of skeletal muscle mass and is associated with a decrease in health and quality of life. As muscle mass declines, the ability to perform physical tasks is greatly reduced, leading to decreased independence and increased factors of morbidity and mortality (37). The study of atrophy and the associated molecular pathways is of great importance to understand this condition and guide research in the development of future therapies.

One cause of skeletal muscle atrophy occurs during prolonged mechanical unloading and disuse of muscle. This can be due to scenarios such as microgravity, sedentary lifestyle, bedrest, decreased physical activity after surgery, limb immobilization, and spinal cord injury. As an example, statistics from a 2010 report indicated 51.4 million surgical procedures were performed at nonfederal hospitals alone in the United States, and the rate of surgical procedures is on the rise (13). Often postoperative instructions to surgery patients include bed rest, reduced physical activity, or body immobilization around the site of injury and can result in abnormally disused muscle and reductions of this tissue over time. Ultimately, these scenarios encompass a lack of mechanical stress and muscle fiber contraction leading, potentially, to profound losses of skeletal muscle and subsequent decreased quality of life and increased health care costs. While interventions exist during and after an atrophic setting, such as rehabilitation methods, the need to develop therapies before entering these settings to maintain skeletal muscle tissue is worth investigation.

Atrophy caused by unloading and disuse is characterized by reductions in size, weight, and function of the tissue (50). When this occurs, molecular changes leading to the net loss of functional protein in muscle tissue are observed. Moreover, previous data indicate correlations between muscle atrophy, mitochondrial dysfunction, and increased oxidative stress (33, 44). It is well established that excessive reactive oxygen species (ROS) observed during muscle atrophy activates protein degradation and cell apoptotic pathways while decreasing protein synthesis resulting in a net protein loss (3, 23, 30). Similarly, past research reveals ROS also damages functional components of the mitochondria leading to reductions in physiologic processes and mitochondrial dysfunction. More specifically, excessive ROS mutates unprotected mtDNA leading to the translation of defective mitochondrial proteins resulting in mitochondrial dysfunction (55).

Exercise is a powerful stimulator of mitochondrial function (45). On the molecular level, exercise activates the master regulator protein of mitochondrial biogenesis, peroxisome proliferator-activated receptor gamma coactivator 1-alpha (PGC-1α), via signaling cascades of acute and immediate changes in circulating and cellular molecules as a part of the exercise response such as epinephrine, growth factors, AMP, and Ca^2+^ (41). This master regulator co-activates with nuclear respiratory factors 1 and 2, increasing the transcription of mitochondrial transcription factor A (TFAM), a well-known marker positively correlated with mtDNA copy number and mitochondrial function (53).

While many studies exist analyzing intra- and postatrophic setting interventions to alleviate atrophy, few studies observe and discuss therapies to prevent these negative changes before an atrophic setting (47, 56). Nakamura et al. (32) found a protective effect of an 8-wk endurance exercise training protocol being completed eight weeks before one-week hindlimb suspension in rat fast-twitch muscle fibers, whereas Fujino et al. (14) found evidence of protective effects after a single-endurance exercise session before hindlimb suspension (HLS). Different types and durations of exercise result in varying adaptations and are worth studying, particularly on a molecular level, to assess these differences. When entering a known atrophy-causing scenario, such as prescribed bed rest or immobilization after elective surgery, being able to intervene physical training or molecularly directed treatments to prevent muscular atrophy could be extremely beneficial for patient quality of life and in reducing financial burdens by mitigating medication and rehabilitation costs.

In a previous review of skeletal muscle atrophy, we proposed a protective effect of exercise before an atrophic setting and potential associated molecular mechanisms (50). Therefore, in the present study we hypothesize exercise training before skeletal muscle unloading and disuse will increase mitochondrial markers and protect skeletal muscle from disuse atrophy. To test this, we use a seven-day HLS protocol to induce muscle atrophy in the hindlimbs of mice compared with mice performing a two-week, concurrent exercise training protocol consisting of endurance running and high-intensity sprinting before seven days of HLS. Results indicate exercise increases markers of mitochondrial biogenesis and function before entering HLS and this preconditioning is associated with the prevention of skeletal muscle atrophy post-HLS. Short-term, concurrent exercise training is attractive for protective therapy to intervene before disuse settings.

## METHODS

### 

#### Animals and ethical approval.

The present study used male C57BL/6J mice. Four groups of mice were used including a control, HLS, exercise before HLS (Ex+HLS), and exercise only (Ex). All group sample sizes consisted of *n* = 6–8 mice total. Mice were killed immediately after HLS treatments and within 48 h of exercise-only treatments. The mice exercising before suspension were placed in HLS during the same day of their last exercise session. Mice entering HLS were 12 ± 1 wk of age, and mice entering the exercise protocol were 10 ± 1 wk of age. All mice were 13 ± 1 wk of age at the time of death. The mice were purchased from Jackson Laboratory (Bar Harbor, ME) and all standard procedures and experiments involving animals conformed with the National Institutes of Health Guidelines and were approved by the Institutional Animal Care and Use Committee of the University of Louisville.

#### Hindlimb suspension.

Mice were suspended by the tail in custom-built cages to unload the hindlimb musculature and induce atrophy for a period of seven continuous days. Cages were constructed as previously shown (11). Briefly, mice were first placed under continuous isoflurane anesthesia, and a harness was fashioned to the tail. The tail was cleaned and surrounded with tape cross-sectionally. A 27-gauge needle cap was cut down, as to be open on each end, to ~2 cm in length. A small hole was drilled into the sidewall of the needle cap, and a piece of nylon string was tied into a loop through this hole. This cap was then placed on the tail and taped into place roughly one-third of the tail length from the base. The nylon loop could then be attached to the roof of the cage, suspending the animal’s hindlimbs while allowing the forelimbs to bear weight and the animal to move around the cage. Mice could also access food and water ad libitum in this manner. Body weights were recorded before and after suspension.

#### Exercise protocol.

Exercise consisted of 14 sessions over 18 days of treadmill running in a concurrent exercise program (i.e., combining different exercise styles in the same program). Mice were acclimated to the treadmill during the first four sessions (*week 1*). The subsequent 10 sessions (*weeks 2* and *3*) consisted of six endurance training style exercise sessions and four high-intensity interval style sprint exercise sessions. Sprint exercise consisted of alternating between high-intensity sprints and low-intensity walking speeds throughout a single session. Complete rest was given every 100 m during endurance training and after every five sprints during high-intensity training. All exercise program details (Table 1) were matched between exercising groups.

**Table 1.** Exercise protocol

	Monday	Tuesday	Wednesday	Thursday	Friday
*Week 1*					
Warm-up		7 m/min for 50 m	7 m/min for 50 m	7 m/min for 50 m	7 m/min for 50 m
Work rate	off	7 m/min	8 m/min	9 m/min	10 m/min
Rest		3 min off/100m	3 min off/100 m	3 min off/100 m	3 min off/100 m
Distance		300 m	300 m	300 m	300 m
*Week 2*	Monday	Tuesday-Sprints	Wednesday	Thursday-Sprints	Friday
Warmup	7 m/min for 50 m	7 m/min for 50 m	7 m/min for 50 m	7 m/min for 50 m	7 m/min for 50 m
Work Rate	11 m/min.	15 m/min for 10 m	12 m/min	17 m/min for 15 m	13 m/min
Walk Rate		7 m/min for 10 m		7 m/min for 15 m	
No. of Sprints		8		10	
Rest	3 min off/100 m	5 min after 5 sprints	3 min off/100 m	5 min after 5 sprints	3 min off/100 m
Distance	350 m	160 m	375 m	300 m	400 m
*Week 3*	Monday	Tuesday-Sprints	Wednesday	Thursday-Sprints	Friday
Warmup	7 m/min for 50 m	7 m/min for 50 m	7 m/min for 50 m	7 m/min for 50 m	7 m/min for 50 m
Work Rate	13.5 m/min	18 m/min for 15 m	14 m/min	20 m/min for 20 m	14.5 m/min
Walk Rate		7 m/min for 15 m		5 × −7 m/20 m; 6 × 7 m/10 m	
No. of Sprints		11		11	
Rest	3 min off/100 m	5 min after 5 sprints	3 min off/100 m	5 min after 5 sprints	3 min off/100 m
Distance, m	450	330	500	380	550

Mouse concurrent treadmill exercise protocol consisting of a *week 1* acclimation, followed by progressive training *week 2* and *week 3*. Three days per week (M, W, F) consisted of endurance-style exercise, whereas two days per week (T, TH) consisted of higher-intensity interval sprint style of exercise.

#### Laser doppler imaging.

Lower limb blood flow was measured for each group using a laser doppler imaging system (Moor FLPI, Wilmington, DE) to assess flux of the hindlimb at the end of each treatment and just before death. Mice were first administered tribromoethanol based on body weight. The musculature of the left lower hindlimb was then exposed, revealing the musculature of the leg. Each mouse was placed in a prone position and the laser was positioned 15 cm from the area of interest. The laser was site directed to the posterior tibial vein, which is a prominent vessel on the posterior hindlimb located superficial to the gastrocnemius, allowing an accurate reading with our laser. Recordings of flux were measured for two minutes and quantified.

#### Tissue extraction and muscle weight.

Soleus and gastrocnemius tissues were excised and separated from each leg in all experimental groups, washed with 50 mmol/l phosphate-buffered saline (PBS) pH of 7.4, weighed individually, snap-frozen in liquid nitrogen, and stored at −80°C until use. Muscle weights reported were the average between the left and right muscle. All methods and timings of tissue extraction were equated across all groups.

#### Immunofluorescence.

A portion of each muscle was cut cross-sectionally at the mid belly and immersed in tissue-freezing medium (Triangle Biomedical Sciences, Durham, NC) in disposable plastic tissue-embedding mold (Polysciences Inc., Warrington, PA). The tissue blocks were immediately frozen and kept at −80°C until use. Sections 7–10 µm thick were obtained from each muscle using a Cryocut System (model CM 1850; Leica Microsystems, Buffalo Grove, IL). Tissue sections were placed on poly-l-lysine-coated microscope slides (Polysciences, Inc.). Tissue sections were fixated in acetone for 20 min, washed in 1× PBS-T solution, and incubated with permeabilization solution (0.2 g BSA, 3 µl Triton X-100 in 10 ml 1× PBS) for 1 h at RT followed by another washing step with 1× PBS-T. The sections were incubated with primary antibody (anti-Laminin; Abcam, Cambridge, MA) dilution of 1:200 overnight at 4°C. After washing with 1× PBS-T the slides were incubated with a fluorescently labeled secondary antibody (goat anti-mouse Alexa Fluor 594; Invitrogen, Waltham, MA) with a 1:300 dilution for 1 h at RT. After another washing step, slides were mounted with mounting medium and glass coverslips, and visualized using a laser scanning confocal microscope (Olympus FluoView 1000; Center Valley, PA). Cross-sectional area measurements were acquired using laminin images by hand tracing single stained muscle fibers in ImageJ software (43).

#### Dihydroethidium staining for oxidative stress.

After excision, each muscle was cross-sectioned in half, immediately placed in tissue-freezing medium (Triangle Biomedical Sciences, Durham, NC), snap-frozen and stored at −80°C until use. Samples were then sectioned on a cryostat, placed on slides, and in situ superoxide generation was evaluated in each cryosection with the oxidative fluorescent dye dihydroethidium (DHE). Cryosections (10 μm) were incubated with DHE (2 μmol/l) in PBS. After washing, slides were mounted with mounting medium and a coverslip, and then visualized using a laser scanning confocal microscope (Olympus Fluo View 1000; Center Valley, PA).

#### Protein extraction and protein estimation.

Muscle tissue samples were bead-homogenized in ice‐cold RIPA (1 mmol/l) buffer with PMSF and protease inhibitor cocktails (1 μl/ml of lysis buffer, Sigma Aldrich, St. Louis, MO). The samples were then centrifuged at 12,000 g for 20 min at 4°C. The supernatant was extracted and stored at −80°C until use. Protein estimation was measured by the Bradford‐dye (Bio‐Rad, CA) method in a 96‐well microliter plate against a BSA standard. The plate was analyzed at 594 nm in a Spectra Max M2 plate reader (Molecular Devices Corporation, Sunnyvale, CA).

#### GSH/GSSG ratio for redox status.

To quantify oxidative stress, a GSH/GSSG ratio detection assay kit (Abcam) was used as previously described (46). Briefly, protein lysates were first deproteinized by using trichloroacetic acid, incubated on ice (15 min), and centrifuged (5 min). All supernatant was removed and neutralized using the stock neutralizing solution (Abcam). The assay was performed against GSH and GSSG standards. Samples were incubated and monitored between 10 and 60 min and analyzed on 96-well plates at 490/520 nm wavelengths in a Spectra Max M2 plate reader (Molecular Devices Corporation, Sunnyvale, CA). All samples were loaded in duplicate.

#### Western blot analysis.

Protein lysates (40 µg) were prepared and heated at 95°C for five min and loaded in an SDS polyacrylamide gel in running buffer and run at a constant current (75 volts). Proteins were then transferred to a PVDF membrane overnight at 120 mA at 4°. After transfer, the membranes were blocked in 5% nonfat milk for 1 h at room temperature followed by overnight incubation with primary antibodies (anti-PGC-1α, anti-TFAM, anti-SMHC, anti-CS, anti- MTCO1, anti-HSP60, anti-SOD2; Abcam, Cambridge, MA) at 4°C. After washing with TBS-T buffer, membranes were incubated with secondary antibodies (horseradish peroxidase-conjugated goat anti-mouse, goat anti-rabbit IgG; Santa Cruz Biotechnology, Dallas, TX) for 1 h at RT with 1:5,000 dilution followed by washing. The membranes were developed with an ECL Western blotting detection system (GE Healthcare, Piscataway, NJ), and all images were recorded in the gel documentation system ChemiDoc XRS (Bio-Rad, Richmond, CA). The membranes were stripped with stripping buffer (Boston BioProducts, Ashland, MA) followed by a blocking step with 5% milk for 1 h at RT. After being washed, membranes were incubated with anti-Gapdh antibody (Millipore, Billerica, MA) as a loading control protein. Peroxisome proliferator-activator receptor gamma coactivator-1α (PGC-1α) and slow myosin heavy chain (SMHC) were visualized on the same gels; mitochondrial transcription factor A (TFAM) and citrate cynthase (CS) were visualized on the same gels; and heat shock protein 60 (HSP60) and mitochondrial complex I (MTCO1) were visualized on the same gels due to size similarities. The data were analyzed by Bio-Rad Image Laboratory densitometry software and normalized to anti-Gapdh bands.

#### Quantitative PCR.

To assess mRNA expression of different genes in skeletal muscle, RNA was isolated with TRIzol reagent (Life Technologies, Carlsbad, CA) according to manufacturer's instructions. The RNA quantification and purity were assessed by NanoDrop-1000 (Thermo Scientific, Walthan, MA). Aliquots (2 μg) of total RNA were reverse-transcribed into cDNA using a high-capacity cDNA reverse transcription kit (Applied Biosystems, Foster City, CA) according to the manufacturer’s protocol. q-PCR was performed for seven different genes (PGC-1α, TFAM, CS, Myh4, Myh7, SOD-1, and SOD-2) in a final reaction volume of 20 µl containing 10 µl of PerfeCTa SYBR Green SuperMix, Low ROX (Quanta Biosciences, Gaithersburg, MD), 6 µl nuclease-free water, and 2 µl cDNA, 40 pmols of forward and reverse primers. All sequence-specific oligonucleotide primers (Invitrogen, Carlsbad, CA) are presented in (Table 2). The data reported from q-PCR is represented in fold expression calculated as the cycle threshold difference between control and sample and normalized with the housekeeping genes beta actin and 18s.

**Table 2.** qPCR primers

Gene	Forward Nucleotide Sequence	Reverse Nucleotide Sequence
Myh7	5′-ATCAAATCATCCAAGCCAACCC	5′-GAGGAGTTGTCATTCCGAACTG
Myh4	5′-TTGTGGTGGATGCTAAGGAGTC	5′-GTACTTGGGAGGGTTCATGGAG
CS	5′-CACTGTTGAGATGGACACACTG	5′-TCCTGAAGTCTGCATCATGACT
SOD-1	5′-GAACCAGTTGTGTTGTCAGGAC	5′-GCCTTGTGTATTGTCCCCATAC
SOD-2	5′-GGTCACAGTTTCACAGTACACC	5′-TCACAGCCTTGAGTTACAGAGT
TFAM	5′-GAGAGCTACACTGGGAAACCACA	5′-CATCAAGGACATCTGAGGAAAA
PGC-1a	5′-CTTTCTGGGTGGATTGAAGTGG	5′-CTCAAATATGTTCGCAGGCTCA
Rn18S	5′-cacggacaggattgacagattg	5′-gacaaatcgctccaccaactaa
β-actin	5′-ccctgaagtaccccattgaaca	5′-cacacgcagctcattgtagaag

Nucleotide sequences of all examined genes. Myh, myosin heavy chain; CS, citrate cynthase; SOD, superoxide dismutase.

#### Statistical analysis.

All data are means ± SE. One-way ANOVA analysis was conducted on each data set with Tukey’s post hoc analysis used between groups after significance was obtained. Significance was determined as a *P* < 0.05.

## RESULTS

### 

#### Exercise diminishes loss of muscle weight and cross-sectional area.

**Fig. 1.**
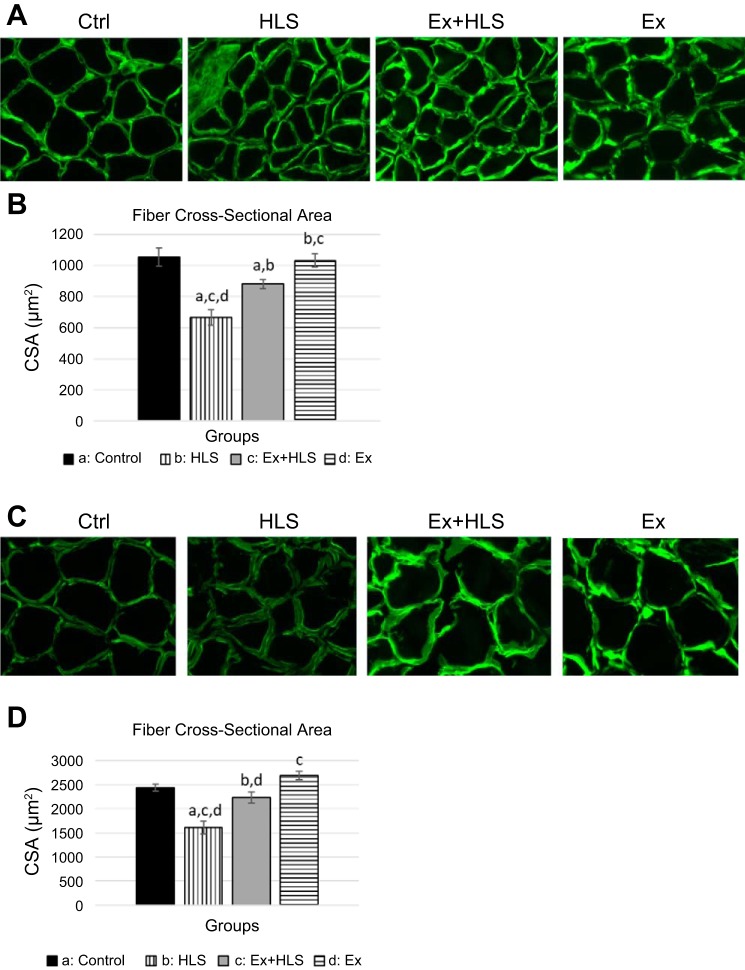
*A*: immunofluorescence laminin staining of soleus muscle fibers (200 µm width). HLS, hindlimb suspension; Ex, exercise. *B*: cross-sectional area (CSA) measurement acquired using ImageJ software (*n* = 6). *C*: immunofluorescence laminin staining of gastrocnemius muscle fibers (200 µm width). *D*: CSA measurement acquired using ImageJ software (*n* = 6). Statistical significance (*P* < 0.05, means ± SE) indicated by the corresponding group letter.

Wet muscle weight was measured immediately after excision. Muscle weights were standardized to body weight (mg/g) to account for individual size variation (Table 3). HLS resulted in a significant decrease (*P* = 0.001) of 27.1% in soleus muscle and a significant decrease (*P* = 0.001) of 21.5% in gastrocnemius muscle weight-to-body weight ratio compared with control. Exercising before HLS resulted in only a 5.6% nonsignificant decrease (*P* = 0.50) in soleus muscle and an 8.1% nonsignificant decrease (*P* = 0.16) in gastrocnemius weight-to-body weight ratio (Table 3) compared with control. Both the soleus and gastrocnemius Ex+HLS group ratios were significantly greater than the HLS group (*P* = 0.001 and *P* = 0.007, respectively). Furthermore, cross-sectional area (µm^2^) differences were similar with HLS resulting in a significant decrease compared with control (*P* < 0.01), whereas Ex+HLS and Ex groups were both significantly greater than HLS (*P* < 0.01) (Fig. 1).

**Table 3.** Body weight and muscle weight

Group	Pre-HLS Body Wt, g	Body Wt, g	Sol MW, mg	Gas MW, mg	Sol MW/Body Wt, mg/g	Gas MW/Body Wt, mg/g
Control	—	24.5 ± 0.55	7.8 ± 0.17	121.1 ± 6.1	0.32 ± 0.007	4.9 ± 0.164
HLS	23.3 ± 0.87	22.0 ± 0.79	5.1 ± 0.15^a^^,^^c^^,^^d^	85.4 ± 5.5^a^^,^^c^^,^^d^	0.23 ± 0.008^a^^,^^c^^,^^d^	3.9 ± 0.236^a^^,^^c^^,^^d^
Ex+HLS	24.3 ± 0.78	22.4 ± 0.67	6.9 ± 0.33^a^^,^^b^^,^^d^	103.8 ± 4.3^a^^,^^b^	0.30 ± 0.012^b^^,^^d^	4.5 ± 0.065^b^
Ex	—	23.5 ± 0.22	7.9 ± 0.12	110.7 ± 4.3	0.34 ± 0.003	4.7 ± 0.151

Values are means ± SE. Body Wt, body weight, pre-HLS, pre-hindlimb suspension, and Body Wt at time of death. MW, muscle weight of both the soleus (Sol) and gastrocnemius (Gas). Value is average of left and right muscle weights. Ex, exercise. Statistical significance (*P* < 0.05) between groups indicated by the superscript letter associated with each particular group:

a control,

b HLS,

c Ex+HLS, ^d^Ex.

#### Hindlimb blood flow is not a limiting factor.

**Fig. 2.**
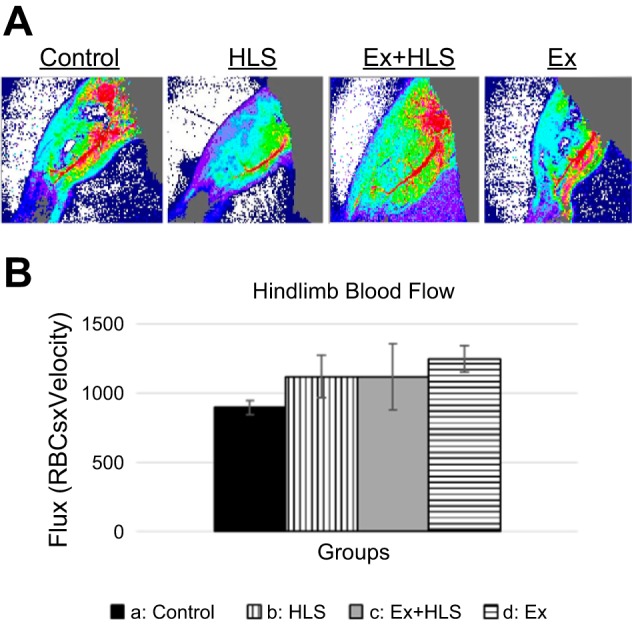
*A*: laser Doppler blood flow measurement of prominent vessel in the dorsal hindlimb. *B*: blood flux measurement comparison between groups (*n* = 6, means ± SE). HLS, hindlimb suspension; Ex, exercise.

Laser doppler imaging of the left hindlimb assessed blood flow through the posterior tibial vein, superior to the gastrocnemius (Fig. 2). This measurement is a function of red blood cell (RBC) content and the velocity of RBCs through the vessel of interest. Here we show that while Ex tended to increase blood flow in this particular vessel there were no significant differences between groups (*P* = 0.53).

#### Exercise increases functional muscle protein.

**Fig. 3.**
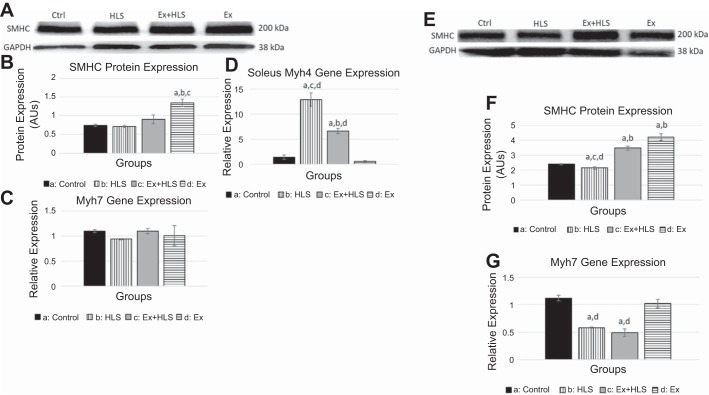
*A*: soleus slow myosin heavy chain (SMHC) protein expression (*n* = 6). HLS, hindlimb suspension; Ex, exercise. *B*: Western blot analysis of SMHC. Statistical significance (*P* < 0.05) between groups indicated by the corresponding group letter. AU, arbitrary units. *C*: myosin heavy chain 7 (MHC7; slow-twitch muscle) gene expression analysis by qPCR. *D*: myosin heavy chain 4 (MHC4; fast-twitch muscle) gene expression analysis by qPCR. *E*: gastrocnemius SMHC protein expression. *F*: Western blot analysis of gastrocnemius SMHC. Statistical significance (*P* < 0.05) between groups indicated by the corresponding group letter. *G*: MHC7 (slow-twitch muscle) gene expression analysis by qPCR. (groups: *n* = 6, *P* < 0.05, means ± SE).

According to sliding filament theory, skeletal muscle contraction results from the cyclic interaction of actin and myosin molecules and the recycling of ATP (18). Loss of these protein molecules is an indicator of muscle atrophy and correlates with decreased fatigue resistance of the muscle (10, 20). HLS resulted in a no significant changes in SMHC protein expression in the soleus muscle and a significant decrease in the gastrocnemius muscle (Fig. 3). However, exercise before HLS resulted in a significant increase in SMHC in the gastrocnemius compared with the HLS group (*P* < 0.05). Furthermore, exercise only significantly increased SMHC compared with controls in soleus (*P* = 0.014) and gastrocnemius (*P* < 0.01). Using qPCR, we followed up protein expression of SMHC with gene expression analysis of a SMHC gene, Myh7. In the soleus, Myh7 approached a significant decrease (*P* = 0.056) in HLS compared with control (0.94 vs. 1.1, respectively). In the gastrocnemius, Myh7 was significantly decreased in both HLS and Ex+HLS compared with control and Ex with no difference being observed in the Ex group transcripts compared with control (Fig. 3).

Moreover, disuse of skeletal muscle and cessation of exercise training is associated with fiber-type transitioning from a slow, more oxidative muscle to a fast, more glycolytic muscle fiber-type composition (4). We, therefore, assessed the transcript levels of the faster, more glycolytic type IIb myosin heavy chain through qPCR analysis of the Myh4 gene. As expected, in soleus tissue HLS resulted in a significant increase in Myh4 mRNA compared with control (21.34 vs 1.15, *P* < 0.01). Although Ex+HLS Myh4 transcript levels were significantly elevated from control, results indicated less mRNA expression of Myh4 compared with HLS (*P* < 0.01) (Fig. 3).

#### Mitochondrial markers increase with exercise.

Markers of mitochondrial biogenesis and function were measured via qPCR and Western blotting analyses to assess our hypothesis that exercise increases these markers which are correlated with the protection of muscle weight and size during HLS. To test this correlation, we assessed the transcript and protein expression of the PGC-1α, TFAM, HSP60, MTCO1, and CS.

**Fig. 4.**
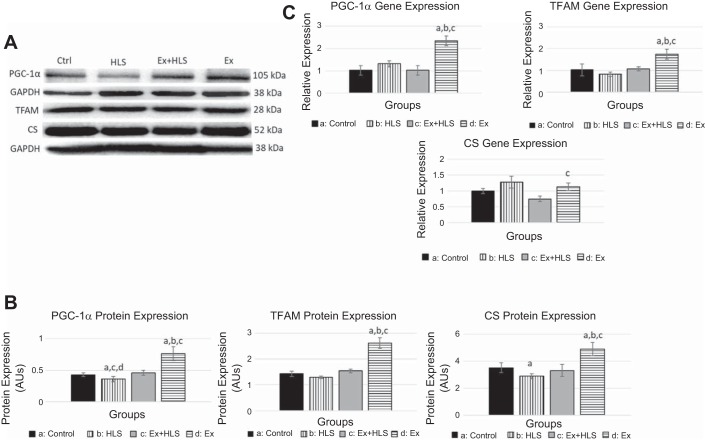
*A*: soleus protein expression via Western blot analysis of mitochondrial markers (SMHC/PGC-1α and TFAM/CS expression measured on same blots with same loading control, due to size similarities). TFAM, mitochondrial transcription factor A; CS, citrate cynthase; HLS, hindlimb suspension; Ex, exercise. *B*: Western blot analysis of soleus muscle mitochondrial markers. Statistical significance (*P* < 0.05) between groups indicated by the corresponding group letter. AU, arbitrary units. *C*: gene expression analysis by qPCR of mitochondrial markers. (groups: *n* = 6, *P* < 0.05, means ± SE).

**Fig. 5.**
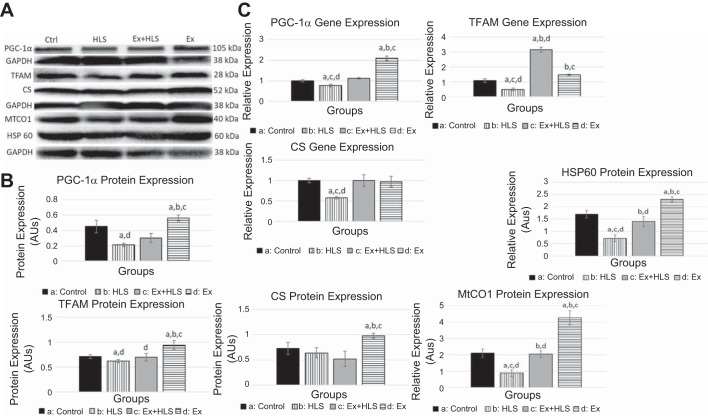
*A*: gastrocnemius protein expression via Western blot of mitochondrial markers (SMHC/PGC-1α and TFAM/CS expression measured on same blots with same loading control, due to size similarities). TFAM, mitochondrial transcription factor A; CS, citrate cynthase; HSP60, heat shock protein 60; MTCO1, mitrochondrial complex 1; HSP60, heat shock protein 60; HLS, hindlimb suspension; Ex, exercise. *B*: Western blot analysis of soleus muscle mitochondrial markers. AU, arbitrary units. Statistical significance (*P* < 0.05) between groups indicated by the corresponding group letter. *C*: Gene expression analysis by qPCR of mitochondrial markers. (groups: *n* = 6, *P* < 0.05, means ± SE).

**Fig. 6.**
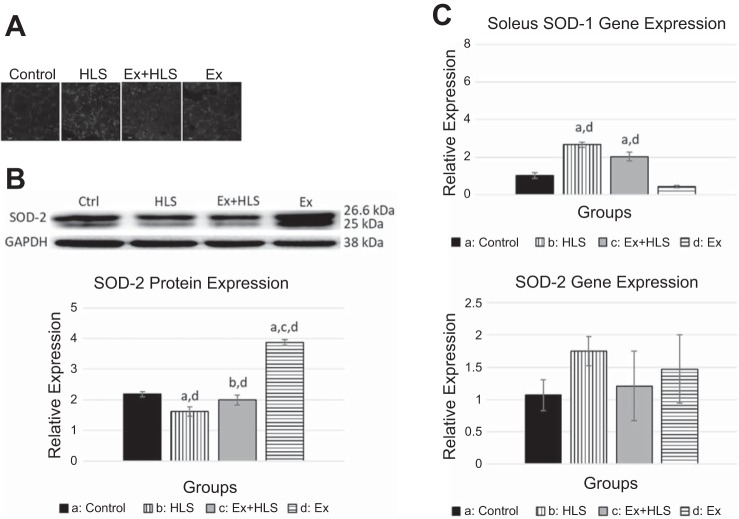
*A*: observational soleus dihydroethidium staining for ROS. Bright spots are assumed to be positive staining for superoxide anion (*n* = 4). HLS, hindlimb suspension; Ex, exercise. *B*: soleus protein expression via Western blot analysis for antioxidant enzyme superoxide dismutase-2 (SOD-2). Statistical significance (*P* < 0.05) between groups indicated by the corresponding group letter. *C*: soleus SOD-1 and SOD-2 gene expression via qPCR. (groups: *n* = 6, *P* < 0.05, means ± SE).

In assessing mitochondrial biogenesis, we measured PGC-1α transcript expression to be significantly increased after the exercise protocol (Ex) in both soleus (Fig. 4) and gastrocnemius muscles (Fig. 5) compared with control (2.3-fold and 1.6-fold, respectively). PGC-1α mRNA levels were significantly decreased in the gastrocnemius after HLS. The Ex+HLS group, although lower than Ex, was still significantly greater than HLS in the gastrocnemius (1.12 vs 0.87, *P* = 0.004). Protein expression of PGC-1α in the soleus muscle (Fig. 6) was significantly greater in Ex compared with control (*P* < 0.05), whereas HLS was significantly reduced (*P* < 0.05). Ex+HLS expression of PGC-1α was significantly greater than HLS alone (*P* < 0.05). In gastrocnemius muscle (Fig. 5), the Ex group revealed a nearly two-fold increase in protein expression (*P* < 0.01). Furthermore, in gastrocnemius muscle HLS decreased PGC-1α compared with control (*P* < 0.01).

TFAM transcript expression significantly increased in the soleus (Fig. 4) after the exercise protocol (Ex) compared with control (1.74 vs 1.03 [*P* < 0.05]). TFAM mRNA expression levels in the gastrocnemius significantly decreased after HLS (*P* = 0.016), with the Ex+HLS group being significantly greater than HLS (3.16 vs 0.49, *P* = 0.001). Protein expression of TFAM was found to be elevated in Ex compared with control in both muscles (*P* < 0.05). HLS only resulted in a significant decrease in the gastrocnemius (*P* < 0.05) (Fig. 5) compared with control, whereas TFAM of Ex+HLS was elevated significantly compared with HLS only in the gastrocnemius (*P* < 0.05).

**Fig. 7.**
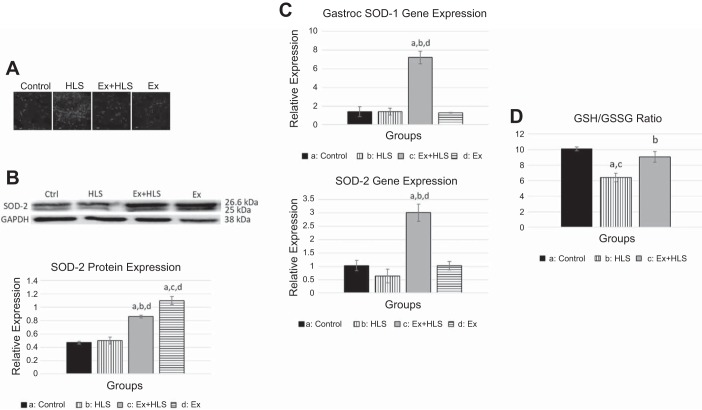
*A*: observational gastrocnemius dihydroethidium staining for ROS. Bright spots are assumed to be positive staining for superoxide anion (*n* = 4). HLS, hindlimb suspension; Ex, exercise. *B*: gastrocnemius protein expression via Western blot analysis for antioxidant enzyme SOD-2. Statistical significance (*P* < 0.05) between groups indicated by the corresponding group letter. SOD-2, superoxide dismutase-2. *C*: SOD-1 and SOD-2 gene expression via qPCR. *D*: GSH/GSSG ratio indicating oxidative stress. (groups: *n* = 6, *P* < 0.05, means ± SE).

Citrate synthetase is a tricarboxylic acid cycle enzyme catalyzing citrate from acetyl-CoA and oxaloacetate. It is a commonly used biomarker to assess mitochondrial function and density in skeletal muscle (54). Analysis of the mRNA expression in the soleus (Fig. 6) revealed no differences between control, HLS, and Ex groups. Soleus CS protein expression (Fig. 6) decreased in the HLS group compared with controls (*P* < 0.05). Moreover, exercise increased CS protein in soleus muscle compared with all other groups (*P* < 0.05). In the gastrocnemius muscle (Fig. 7), CS mRNA transcripts were significantly reduced in HLS compared with control (0.58 vs. 1.01, *P* = 0.0001). Ex+HLS and Ex groups were not significantly different compared with control but were both significantly greater than HLS (*P* < 0.05). Gastrocnemius protein expression of CS (Fig. 7) increased significantly in the Ex group compared with all other groups. However, there were no significant differences between control, HLS, and Ex+HLS.

Heat Shock Protein 60 and MTCO1 were also analyzed for protein expression in the gastrocnemius. HSP60 was significantly greater in Ex+HLS compared with HLS in the gastrocnemius (*P* < 0.05), and MTCO1 similarly was significantly greater in Ex+HLS compared with HLS (*P* < 0.05) (Fig. 5).

#### Exercise before HLS may alter redox status.

Reactive oxygen species are implicated in the induction of protein degradation and apoptotic pathways, potentially being an important contributor to the atrophic muscular condition (3). To visualize ROS accumulation, we used a DHE staining, solely as an observational assessment. DHE staining is commonly used to visualize ROS and in previous research is viewed as the least problematic and most specific ROS dye, due to its ability to easily permeate cell membranes and its specificity for superoxide anion (35). Observationally, there were clear differences between groups (Figs. 6 and 7). HLS, as expected, resulted in greater levels of ROS in both muscles. Ex+HLS appears to be reduced compared with the excessive positive staining for superoxide anion in the HLS group. Additionally, the gastrocnemius GSH/GSSG ratio was conducted as a secondary assessment to quantify oxidative stress in this tissue. Ex+HLS was significantly greater than HLS (*P* < 0.05) and was not significantly different than control (*P* = 0.53) (Fig. 7).

Superoxide dismutases (SODs) are the main antioxidant defense system against superoxide anions (15). Therefore, we measured SOD-1 and SOD-2 (mitochondrial SOD) transcripts via qPCR and SOD-2 protein expression via Western blot to assess any differences between groups with these antioxidants. In the soleus we observed a significant increase in SOD-2 protein expression after exercise (Ex) (*P* < 0.01) compared with all other groups. Gastrocnemius SOD-1 and SOD-2 mRNA expression of the Ex+HLS group was significantly greater than all other groups (*P* < 0.01). SOD-2 protein expression in this muscle was significantly greater in both Ex and Ex+HLS groups compared with both control (*P* < 0.01) and HLS (*P* < 0.01), respectively.

## DISCUSSION

Skeletal muscle atrophy is the result of an imbalance of protein degradation and protein synthesis. When degradation exceeds synthesis, the result is a loss of muscle mass. An atrophy rate of ~0.5% of total muscle mass per day has been reported in the first two to three weeks under disuse atrophy conditions (57). This rate of atrophy can result in debilitating effects, reducing patient health and quality of life.

The correlation between skeletal muscle atrophy and reduced mitochondrial biogenesis and function (38, 39), as well as increased or unbalanced reactive oxygen species accumulation, is well demonstrated in the literature (9, 33). Furthermore, a wealth of research reveals exercise training increases markers of mitochondrial function and biogenesis (16, 26, 29). While treatments exist, intra- or postatrophic settings, the evidence for treatments preatrophic settings is scarce. Therefore, the aim of this study is to assess the effects of preconditioning the muscle tissue with a short-term exercise protocol before hindlimb unloading as a treatment to prevent atrophy. Secondarily, we aim to analyze mitochondrial molecular markers as mechanisms associated with the protective therapy of exercise preconditioning.

Initially, Doppler laser technology used on the hindlimbs of all mice groups under anesthesia assessed blood flow. Differences in oxygen and nutrient delivery as well as waste removal in the muscle tissue via changes in blood flow could account for differences in tissue health. Prior research in this area reveals a disconnect in blood flow assessment and atrophy. Nevertheless, according to a 2001 review of microvasculature changes with disuse atrophy by Tyml et al. (52), multiple animal studies reveal no changes in blood flow in atrophied muscle as well as no differences in mean blood pressure and vascular resistance. Similarly, our measurements reveal no significant differences between all groups, indicating these tissue changes are not RBC and velocity related. Here, we did not account for capillarization and global microvasculature of each muscle, given our laser was solely directed at a more prominent vessel of the gastrocnemius due to the constraints of this particular technology. Furthermore, the nature of blood flow through the lower limb posterior tibial vein may be different in the measured prone position compared with that which occurs during HLS, as a tilted, head-down body position may lead to lower blood flow in the raised hindlimbs. These assessments may account for group differences and are limitations of the present study warranting future consideration.

In muscle-disuse models, previous research assesses muscle atrophy via changes in muscle weight, cross-sectional area, and fiber-type transitioning. Atrophy may occur in as little as three days (2), whereas other studies reveal atrophy occurring after seven or more days of disuse (19, 24, 39). In the present study, we similarly observe muscle atrophy occurring after seven days of HLS in both the soleus and gastrocnemius muscles, indicated initially by reductions in the weight and size of the muscle.

Numerous studies have also intervened various treatments to reduce the effects of atrophy on skeletal muscles including electrical stimulation, nutrition, and exercise intra- or post-HLS (1, 17, 19, 34, 49). However, studies specifically measuring the effects of interventions, particularly exercise training, pre-HLS are scarce. Here we report greater muscle weight and cross-sectional area along with decreased slow-to-fast fiber-type transitioning in mice exercising before HLS compared with mice undergoing only HLS. Similar results of muscle weight and size were observed in a 2016 study in which rats were treated with four months of swimming exercise before having their hindlimbs immobilized for five days in orthosis to induce atrophy (36). Although many differences exist between these studies, the researchers report exercise training before immobilization preserves soleus muscle mass compared with rats without exercise training.

Fujino et al. (14) measured soleus muscle mass after treating rats with one session of endurance exercise before HLS, and, although the exercise group was greater than the suspension group, no significant differences were determined. This group also noted reductions in MHC I, a slow MHC, after 14 days of HLS, whereas the group exercising before HLS was not significantly greater than the HLS group. However, a single exercise session may not be enough stress to induce adequate adaptive changes to provide significant protection of the muscle over an HLS protocol. Furthermore, adapting to exercise requires time and can be repetitiously surmounted to achieve, incrementally, greater adaptations. Such is the foundation of progressive exercise training (51). The use of a single session negates the factor of time to allow incremental increases in adaptation and potentially greater skeletal muscle protection. However, shorter-term exercise programs may be more translatable and useful in human adherence. Therefore, we used a concurrent protocol in this study that combines moderate (endurance running) and high-intensity (sprint running) adaptive stressors over a shorter period (14 sessions) while still allowing enough time for adaptations to surmount. This protocol was specifically chosen to derive the known mitochondrial benefits of endurance training with the stress of completing high-force activity, such as sprint exercise training, to attempt to activate the spectrum of muscle fiber types in both muscles of the present study. We conducted protein expression analysis of fast myosin heavy chain expression but were unable to observe significance between groups due to large variation. However, our results indicated similar SMHC results as the Fujino et al. (14) study in the soleus muscle between these groups. We also report a significant increase in gastrocnemius SMHC in the group exercising before HLS, potentially revealing muscle group differences in exercise adaptations and HLS effects.

Prior research indicates slow muscle fibers, particularly of the soleus muscle, are more susceptible to atrophy during disuse conditions, such as spaceflight and bed rest (56). This may be due to the soleus being a postural muscle and subjected to more prolonged tension with daily activity. Unloading this tissue of the typical, greater tension exposure could result in more pronounced effects of disuse-induced atrophy observed previously. Both the soleus and gastrocnemius in the HLS group weighed significantly less and had significantly lower cross-sectional areas compared with controls in the present study. However, the soleus muscle of the Ex+HLS group was significantly lower than control, but the gastrocnemius was not different than controls in cross-sectional area measurements, indicating a mix of similarities and differences in results with prior literature. The length of time and type of disuse studied throughout the literature may play larger roles in the fiber-type atrophy specificity and should be considered when reviewing this data.

It is also well-established that a reduction in markers of mitochondrial biogenesis and function are correlated with skeletal muscle atrophy (25, 31, 39). Liu et al. (28) observed diminished mitochondrial respiration before activation of protein degradation pathways in a toxin-induced mouse atrophy model, and another group (7) observed markers of mitochondrial biogenesis increasing before the regeneration of muscle after atrophy indicating potential timelines of effect of mitochondrial function. The present research similarly revealed significant decreases in PGC-1α in both muscles while significantly decreasing TFAM in the gastrocnemius after HLS. We also observed a significant decrease of CS and MTCO1, commonly used markers of mitochondrial function, in the gastrocnemius (27) establishing evidence for mitochondrial changes being correlated to the atrophy in the present study.

Based on this evidence, exercise was chosen as a preconditioning treatment to prevent disuse-associated atrophy because of the well-known inducing effect it has on mitochondrial biogenesis, particularly PGC-1α and TFAM, and mitochondrial function (22, 29, 42). It should also be noted that while the present study did not investigate the broad array of specific isoforms of PGC-1α, others have noted different isoforms could be involved in different roles in skeletal muscle, such as isoform 4 playing a role in muscle hypertophy (40). Indeed, our measurements of the Ex group mirrored previous research, as we observed increases in gene expression and protein content across both muscles in many of these markers after the exercise protocol. More specifically, increasing expression of signaling pathways of mitochondrial biogenesis while also observing increases in the mitochondrial import protein HSP60 and functional proteins, such as CS and the electron transport chain protein MTCO1, provides substantial evidence on the effect of the present study exercise protocol on the mitochondria. However, here we found an upregulation of HSP60 in the gastrocnemius with our concurrent exercise training, whereas Barone et al. (1a) only found HSP60 upregulation in type I fibers after endurance training. The present study exercise protocol included high-intensity sprinting along with endurance training, potentially leading to greater activation of the gastrocnemius muscle and type II fibers in this musculature due to the greater force requirements of the training. This greater activation of the gastrocnemius may have led to the differences in HSP60 response.

Furthermore, it may also be that the increased molecular mitochondrial adaptations achieved by exercise provided enough lasting, anti-atrophic benefits over a seven-day HLS protocol to diminish the negative effects on skeletal muscle, even if the results were no different in multiple markers between HLS and Ex+HLS groups. The accumulated expression of these markers via exercise adaptation may have contributed to the protective effect during muscle disuse over the HLS protocol. Moreover, muscle protein synthesis is a high-ATP consuming process and studies suggest a mitochondrial dysfunction role being at the center of diminished muscle protein synthesis in atrophic conditions due to reduced ATP production (21). Exercise, by itself, is also well known to activate protein synthesis pathways (Akt/mTOR) in skeletal muscle (5). Therefore, by concentrating on treating the mitochondria through exercise we attempt to shift the balance of degradation and synthesis toward a more anabolic tissue status through mitochondrial adaptations.

We also find a wealth of evidence in prior research establishing protein degradation markers upregulated with muscle disuse (8, 19) and ROS as a prominent inducer of protein degradation pathways (ubiquitin-proteosome pathway, FOXO3, NF-κB, murf-1, and atrogin-1/MAF-bx), apoptotic pathways, and mitochondrial dysfunction in skeletal muscle atrophy (3, 23, 30). Experiments quantifying ROS are generally variable, leading our account for ROS through DHE staining to be strictly observational in searching for particular trends between groups. Indeed, we observe high expression of ROS, specifically superoxide anion, in both muscles after HLS, whereas exercising before HLS resulted in lower expression of ROS, indicating a potential connection between ROS and muscle health in the present study. Cannavino et al. (2) reported similar results through DHE staining, showing significant increases in superoxide anion after a seven-day hindlimb suspension in mice. To follow up our DHE observation, we quantified oxidative stress through the measurement of reduced and oxidized glutathione. Similarly, results here matched our DHE observations with Ex+HLS having a greater ratio of reduced/oxidized glutathione, indicating lower oxidative stress with exercise preconditioning. This decreased oxidative stress may lead to lower mutations and degradation of functional components of the muscle cell, particularly the mitochondria, and diminished atrophy. However, our analysis of the muscle-specific E3 ubiquitin ligases muscle-RING finger-1 (MuRF-1) and muscle atrophy F-box (atrogin-1/MAFbx) concluded variable results of these degradation-associated proteins in the present study (data not shown). With variations in the literature of studies solely producing mRNA expression compared with mRNA and protein expression of these particular molecules in different atrophic settings (12), as well as potential timeline of measurement, post-transcriptional, and post-translational regulatory factors possible in the present study, an in-depth analysis into the degradation aspects of this studies treatments should be done in the future.

The mitochondrial respiratory chain is responsible for ATP production but also produces superoxide anion under normal physiologic circumstances, mainly from complex I and complex III (6). It is the excessive and unbalanced accumulation of ROS that leads to deleterious effects of mitochondrial and cellular proteins associated with atrophy. SOD-1 and SOD-2 (mtSOD) are antioxidants that neutralize ROS by accepting the unpaired electron of superoxide anion and converting to hydrogen peroxide, which is then further converted to water in the presence of glutathione peroxidase. Exercise acutely increases ROS leading to the transcription of antioxidants that neutralize oxidative stress caused by increased respiration and results in an adaptation of increased levels of SOD over time to neutralize superoxide anions (48). Here, we also observe increased SOD-2 antioxidant expression in both muscles after the exercise protocol in the present study. The gastrocnemius revealed elevated levels of SOD-2 after Ex+HLS in both the mRNA and protein expression and not only after the exercise protocol (Ex) like that of the soleus. The gastrocnemius may be able to better withstand ROS accumulation during atrophic settings after exercise training due to a greater and more lengthy SOD-2 response. While the soleus muscle revealed increased SOD-2 after exercise, by the time the HLS protocol was over, levels were not significantly different compared with HLS, indicating potential differences among muscle fiber types. The change in antioxidants may potentially provide a protective effect against ROS that accumulates during disuse and further implicates exercise as an effective preconditioning treatment.

### 

#### Conclusions.

Skeletal muscle atrophy is correlated with reduced mitochondrial biogenesis and function along with excessive ROS leading to the activation of protein degradation and apoptotic pathways as well as reductions in protein synthesis. Here we show exercise training before an atrophic setting protects skeletal muscle from HLS-induced atrophy by inducing the potentially protective molecular effects of increased markers of mitochondrial biogenesis and function with increased antioxidant status. Exercise training before an atrophic setting may be a useful intervention to alleviate skeletal muscle atrophy caused by disuse and unloading. Furthermore, mitochondrial directed treatments could be a useful area of study in the prevention of muscle atrophy.

## GRANTS

The study was supported by National Heart, Lung and Blood Institute Grant HL74185 to SCT.

## DISCLOSURES

No conflicts of interest, financial or otherwise, are declared by the authors.

## AUTHOR CONTRIBUTIONS

N.T.T. conceived and designed research; N.T.T., N.J., and G.J.W. performed experiments; N.T.T. analyzed data; N.T.T. interpreted results of experiments; N.T.T. prepared figures; N.T.T. drafted manuscript; N.T.T. and N.J. edited and revised manuscript; N.T.T., N.J., G.J.W., and S.C.T. approved final version of manuscript.
